# Dilated Cardiomyopathy: Phosphorus 31 MR Spectroscopy at 7 T

**DOI:** 10.1148/radiol.2016152629

**Published:** 2016-06-20

**Authors:** Victoria M. Stoll, William T. Clarke, Eylem Levelt, Alexander Liu, Saul G. Myerson, Matthew D. Robson, Stefan Neubauer, Christopher T. Rodgers

**Affiliations:** From the Division of Cardiovascular Medicine, Radcliffe Department of Medicine, University of Oxford Centre for Clinical Magnetic Resonance Research (OCMR), Level 0, John Radcliffe Hospital, Oxford OX3 9DU, England.

## Abstract

**Purpose:**

To test whether the increased signal-to-noise ratio of phosphorus 31 (^31^P) magnetic resonance (MR) spectroscopy at 7 T improves precision in cardiac metabolite quantification in patients with dilated cardiomyopathy (DCM) compared with that at 3 T.

**Materials and Methods:**

Ethical approval was obtained, and participants provided written informe consent. In a prospective study, ^31^P MR spectroscopy was performed at 3 T and 7 T in 25 patients with DCM. Ten healthy matched control subjects underwent ^31^P MR spectroscopy at 7 T. Paired Student *t* tests were performed to compare results between the 3-T and 7-T studies.

**Results:**

The phosphocreatine (PCr) signal-to-noise ratio increased 2.5 times at 7 T compared with that at 3 T. The PCr to adenosine triphosphate (ATP) concentration ratio (PCr/ATP) was similar at both field strengths (mean ± standard deviation, 1.48 ± 0.44 at 3 T vs 1.54 ± 0.39 at 7 T, *P* = .49), as expected. The Cramér-Rao lower bounds in PCr concentration (a measure of uncertainty in the measured ratio) were 45% lower at 7 T than at 3 T, reflecting the higher quality of 7-T ^31^P spectra. Patients with dilated cardioyopathy had a significantly lower PCr/ATP than did healthy control subjects at 7 T (1.54 ± 0.39 vs 1.95 ± 0.25, *P* = .005), which is consistent with previous findings.

**Conclusion:**

7-T cardiac ^31^P MR spectroscopy is feasible in patients with DCM and gives higher signal-to-noise ratios and more precise quantification of the PCr/ATP than that at 3 T. PCr/ATP was significantly lower in patients with DCM than in control subjects at 7 T, which is consistent with previous findings at lower field strengths.

## Abstract (synopsis)

Cardiac phosphorus spectroscopy is demonstrated to be feasible in patients at 7 T, giving higher signal-to-noise ratios and more precise quantification of the phosphocreatine to adenosine triphosphate concentration ratio than at 3 T in a group of 25 patients with dilated cardiomyopathy.

## Introduction

Heart failure is a global health problem that causes widespread morbidity and mortality (1). Heart failure due to dilated cardiomyopathy (DCMdilated cardiomyopathy) is characterized by increased ventricular volume and global impairment of systolic function (2). Phosphorus 31 (^31^P) magnetic resonance (MR) spectroscopy provides unique insight into cardiac energetics in vivo but is a technique with intrinsically low signal-to-noise ratio (SNRsignal-to-noise ratio) because of low metabolite concentrations (3); a low gyromagnetic ratio, γ^31^P; and relatively long T1 relaxation times. These factors lead to undesirable variability in human spectra and impede single-subject comparisons (3–5). ^31^P MR spectroscopic studies in patients with DCMdilated cardiomyopathy have demonstrated derangement of cardiac energetics characterized by a reduction in the phosphocreatine (PCrphosphocreatine) to adenosine triphosphate (ATPadenosine triphosphate) concentration ratio (PCr/ATPphosphocreatine to adenosine triphosphate concentration ratio), which may be superior to the New York Heart Association functional class or left ventricular (LVleft ventricle) ejection fraction for prediction of mortality in patients with DCMdilated cardiomyopathy (6,7).

Whole-body 7-T imagers capable of cardiac MR imaging recently have become available, and ^31^P MR spectroscopy has been shown to be feasible in healthy volunteers at 7 T (8). We hypothesize that these new systems can be used in patients with cardiac disease and that they will allow an improvement in the quality of ^31^P MR spectroscopy, enabling detection of small changes in metabolite concentrations or studies in small patient groups, which will further the understanding of cardiac energetics. Imaging patients with cardiac disease instead of healthy volunteers poses additional challenges such as the potential inability of the patients to tolerate the length of the examination and the physiologic monitoring in the magnet bore, and a potential reduction in the fraction of myocardium within a spectroscopic voxel due to the thinning of the ventricular walls in patients with DCMdilated cardiomyopathy, which may challenge our ability to correct for blood contamination. This study was designed to test whether the increased ^31^P MR spectroscopic SNR at a field strength of 7 T improves precision in cardiac metabolite quantification in patients with DCMdilated cardiomyopathy compared with that with imaging at 3 T.

## Materials and Methods

### Study Cohort

This study was approved by the Solihull ethics committee (REC Ref 13/WM/0155) and all participants gave written informed consent. Patients were eligible for inclusion if they had a clinical diagnosis of DCMdilated cardiomyopathy and an LVleft ventricle ejection fraction of less than 50%, as measured with the Simpson biplane method from echocardiographic data. In total, 101 patients were considered for this study. Patients were excluded if they did not wish to participate (22 patients), if their heart was not beating in sinus rhythm (seven patients had atrial fibrillation), if they had valvular heart disease (five patients), or if they had a contraindication to MR imaging at 3 T or 7 T (21 for implanted cardiac devices or cardiac resynchronization therapy implants, 18 for metal from previous surgery, and three for tattoos). Another exclusion criterion was coronary artery disease, but none of the patients had it. The exclusion criteria for 3-T MR imaging were familiar to the clinical care team already (atrial fibrillation, valvular disease, implantable cardioverter defibrillators or cardiac resynchronization therapy devices), but given the relatively strict screening criteria for 7-T MR imaging, approximately 40% of potential patients who completed the laboratory volunteer screening form were found to have a safety contraindication to MR imaging at 7 T. The LVleft ventricle ejection fraction was verified from the first study sequence, and patients would have been excluded if they had had an LVleft ventricle ejection fraction greater than 50% according to cardiac MR imaging, but no patients did. In total, 25 patients with DCMdilated cardiomyopathy (mean age ± standard deviation, 54 years ± 12, 68% men) were enrolled in the study, and 10 age- and sex-matched healthy control subjects (mean age, 52 years ± 12, 80% men) with no history of cardiac disease were enrolled for ^31^P MR spectroscopy at 7 T only.

### Clinical Measurements

All patients answered the Minnesota Heart Failure questionnaire (9), which is a quality-of-life score with 21 questions used to assess heart failure symptoms over the preceding 4 weeks, with scores ranging from 0 (no effect) to 5 (great effect). Height and weight were recorded, and body mass index was calculated. Blood pressure was recorded (Dinamap-1846-SX; Critikon, Tampa, Fla). Venous blood was drawn for brain-type natriuretic peptide levels. Participants underwent a 6-minute walk test (10).

### ^31^P MR Spectroscopic Protocol

In this study, we compared best-in-class 3-T methods against our newest 7-T hardware and methods to quantify the real-world improvement. Each patient underwent ^31^P MR spectroscopic imaging with a 3-T imager (Trio; Siemens, Erlangen, Germany) and a heart-liver coil (5) and with a 7-T imager (Siemens) and a 16-channel coil (Rapid Biomedical, Würzberg, Germany) (11). The heart-liver coil comprises a 28 × 27–cm^2^ rectangular ^31^P transmit loop (also used for hydrogen 1 [^1^H] transmit and receive) and a loop/butterfly quadrature ^31^P receive pair (12 × 15−cm loop and 23 × 12−cm butterfly) connected through a hardware quadrature combiner to a single receive channel. The 16-channel coil comprises a rigid 26 × 28−cm^2^ rectangular ^31^P transmit element and a flexible set of 16 overlapping 4-cm diameter circular receive elements in a 4 × 4 grid.

Imaging was performed by two operators (V.S., a clinician with 3 years of experience in cardiac MR imaging and either C.T.R. or W.T.C., with 8 and 4 years of experience in ^31^P MR spectroscopy, respectively). The 3-T coil was chosen because, of the coils available in our laboratory, it historically has performed best in vivo (3,5,12,13), and this was confirmed with phantom imaging sequences. (Specifically, at 3 T, the heart-liver coil has a low drop-off in transmit performance throughout the heart [40% drop-off between 8-cm and 12-cm depth vs 64% for a 10-cm loop]; it has a good receive SNR at the depth of the heart [approximately 10 cm], measured as 6% better than that with an eight-element receive array in healthy volunteers [12]; and it benefits from a larger head-to-foot and left-to-right field of view compared with those of smaller loop coils, making coil placement less critical than with a 10-cm loop [8]). At 7 T, the 16-element array was chosen because phantom imaging showed that it gives more uniform transmit performance and greater SNRsignal-to-noise ratio at the depth of the heart (approximately 10 cm), as shown in figure 2 of reference 11. Participants were imaged prone at 3 T (required for the coil) and supine at 7 T (for improved comfort). Both sequences were performed sequentially on the same day to minimize any physiologic variation. Spectroscopic sequences were not gated to avoid potential bias due to mistriggering, particularly at 7 T (14). (Although we note that recent studies in Oxford were not gated at 3 T, and not gating avoids the potential for artifacts from mistriggering, which also often occurs during a 28-min sequence in patients at 3 T). Control subjects underwent ^31^P MR spectroscopy as described at 7 T only, because we already have characterized the performance of the 3-T heart-liver coil extensively (3,5).

As previously described, localization was performed and subject-specific B_1_ maps were computed (5,8). Spectra were recorded by using a chemical-shift imaging pulse sequence (three-dimensional phase-encoded “ultrashort echo time” chemical shift imaging) with matrix, 16 × 16 × 8; voxel size, 15 × 15 × 25 mm^3^; acquisition weighting with 10 averages (k = 0); and repetition time, 1 second (8). At7 T, excitation was at 400 V (ie, 3.2 kW), giving a field of approximately 10 µT in the interventricular septum, and hence, a flip angle of approximately 30° there. At 3 T, flip angles were matched to those at 7 T by using the subject-specific B_1_ maps. Excitation was centered at −250 Hz at 3 T and at more than +266 Hz at 7 T (both relative to PCrphosphocreatine). A 25-mm-thick saturation band suppressed the signal from the anterior chest wall. At 7 T, this was set to the maximum voltage permissible (equivalent to the maximum power permissible) within the radiofrequency heating limits for each subject.

Spectra from a voxel overlying the midventricular septum were fitted by an expert in MR spectroscopy (W.T.C.) under the guidance of another expert in MR spectroscopy (C.T.R.) by using a custom Matlab (Mathworks, Natick, Mass) implementation of the advanced method for accurate, robust, and efficient spectroscopic (AMARES) fitting (15), with prior knowledge specifying 11 Lorentzian peaks (α, β, γ-ATPadenosine triphosphate, PCrphosphocreatine, phosphodiester, and 2 × 2, 3-diphosphoglycerate), fixed amplitude ratios, and literature values for the scalar couplings for the multiplets. This set of prior knowledge has been used successfully in several previous ^31^P MR spectroscopic studies in Oxford, UK (5). The residual after fitting the spectrum typically showed no features above the noise level, suggesting that this is an adequate description of the spectra. We then corrected for blood contamination (16) and partial saturation (17) by using T1 values from the literature (5,8). The final PCr/ATPphosphocreatine to adenosine triphosphate concentration ratio was taken as PCrphosphocreatine/γ-ATPadenosine triphosphate by discounting α-ATPadenosine triphosphate, because it overlaps nicotinamide adenine dinucleotide phosphate (NADPH) and β-ATPadenosine triphosphate because it was not fully excited at 7 T and had a phase artifact in some subjects at 3 T. Finally, the spectral SNRsignal-to-noise ratio was determined (18), and the uncertainty in metabolite concentrations was expressed by their Cramér-Rao lower bounds (19); Cramér-Rao lower bounds give the theoretical minimum for a parameter’s 95% confidence limits.

### Additional Cardiac MR Imaging Sequences

LVleft ventricle volume stacks were recorded for all subjects at 3 T by using a 32-channel cardiac coil to acquire steady-state free precession cine images, which were analyzed by using software (Fusing cmr42; Circle Cardiovascular Imaging, Calgary, Canada) as previously described (20). All patients had an LVleft ventricle ejection fraction less than 50%, which is consistent with their measurements before enrollment. To determine midventricular peak systolic circumferential strain and diastolic strain rate, myocardial tagging was performed (21,22) and analyzed by using software (CimTag2D; Auckland Medical Research, Auckland, New Zealand) (23).

### Statistical Analysis

Statistical analysis was performed with software (SPSS; IBM, Chicago, Ill). Data were tested for normality by using the D’Agostino and Pearson omnibus normality tests and were presented as means ± standard deviation. Two-group comparisons for normally distributed data were analyzed by using the Welch *t* test, or with the paired Student *t* test, where appropriate, while nonnormally distributed data were analyzed with the Mann Whitney *U* test. Correlation was assessed with the Pearson or Spearman correlation coefficient, as appropriate. *P* values less than .05 were considered to indicate a significant difference.

## Results

### Participant Characteristics

Demographic, clinical, and imaging data are shown in Table 1. Although 72% of patients were taking β blockers and 92% were taking angiotensin converting enzyme inhibitors or angiotensin II receptor blockers, there was no significant difference in heart rate or blood pressure compared with control subjects. As expected, the mean LVleft ventricle ejection fraction was significantly lower in patients with DCMdilated cardiomyopathy than in control subjects (35% ± 10 vs 65% ± 3, *P* < .0001), and patients with DCMdilated cardiomyopathy had significantly increased end-diastolic volumes compared with control subjects (295 mL ± 123 vs 164 mL ± 31, *P* < .0001). The peak circumferential systolic strain was significantly impaired in patients compared with control subjects (−10% ± 4 vs −19% ± 2, *P* < .0001) as was the peak diastolic strain rate (45 sec^−1^ ± 22 vs 90 sec^−1^ ± 7, *P* < .0001). Patients with DCMdilated cardiomyopathy had higher blood brain-type natriuretic peptide levels and achieved significantly shorter distances on the 6-minute walk test than did control subjects (see Table 1). Hence the patients with DCMdilated cardiomyopathy recruited to this study had signs of significant LVleft ventricle dysfunction on exertion but remained clinically compensated at rest, supported by a normal resting cardiac output (5.8 L/min ± 1.4).

**Table 1:**
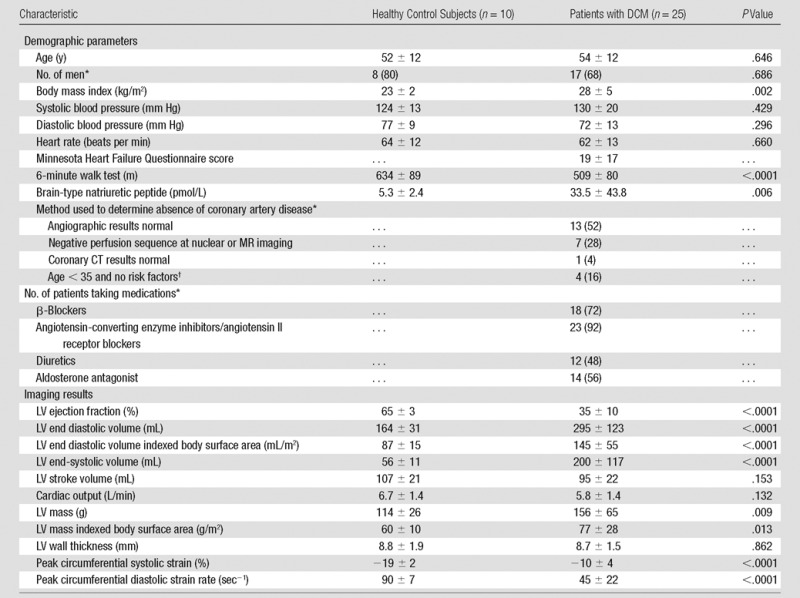
Demographic, Clinical, and Imaging Characteristics of Study Participants

Note.—Unless otherwise indicated, values are means ± standard deviation.

*Data are number of patients, with percentage in parentheses.

^†^For these four patients who were less than 35 years old at the time of diagnosis with no risk factors for coronary disease, a clinical decision was taken not to investigate further because the pretest probability was low.

### ^31^P MR Spectroscopic Results

**Figure 1:**
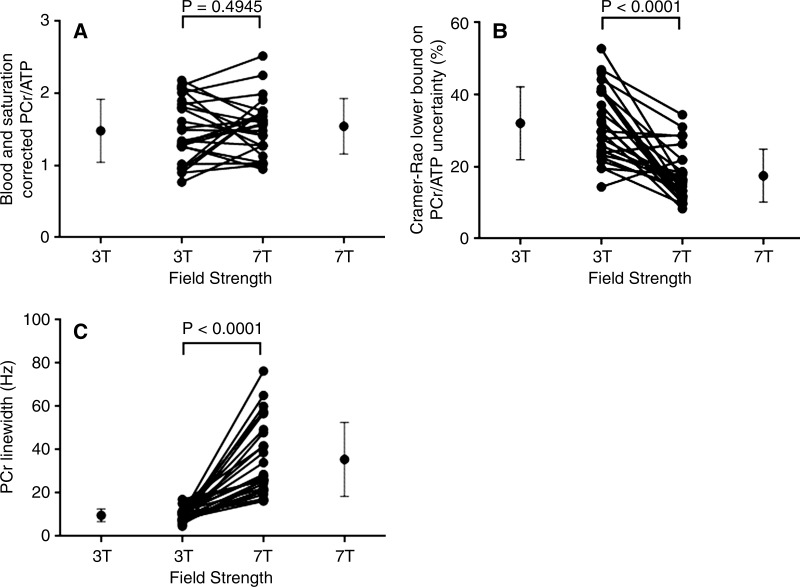
*A*, Graph shows PCR/ATPphosphocreatine to adenosine triphosphate concentration ratio for each patient with DCMdilated cardiomyopathy (mean age, 54 years ± 12; 68% men; further details in Table 1) at 3 T compared with that at 7 T. *B*, Graph shows Cramér-Rao lower bounds for each patient with DCMdilated cardiomyopathy at 3 T compared with that at 7 T. *C*, Graph shows linewidth for PCrphosphocreatine in hertz at 3 T compared with that at 7 T for each patient with DCMdilated cardiomyopathy. Error bars at sides mark mean and standard deviation. In center, each connected pair of points shows data for one patient.

Table 2 summarizes the quantitative ^31^P MR spectroscopic results. As expected, there was no significant difference in the PCr/ATPphosphocreatine to adenosine triphosphate concentration ratio at 7 T and at 3 T (1.54 ± 0.39 vs 1.48 ± 0.44, *P* = .49) for patients with DCMdilated cardiomyopathy, as shown in Figure 1, *A*, and the 7-T PCr/ATPphosphocreatine to adenosine triphosphate concentration ratio for the control subjects (1.95 ± 0.25) was within the accepted range (24). As demonstrated in previous lower-field-strength studies (6,7,25) the PCr/ATPphosphocreatine to adenosine triphosphate concentration ratio was significantly lower, by 21%, in patients with DCMdilated cardiomyopathy than in control subjects (1.54 ± 0.39 vs 1.95 ± 0.25, *P* = .005) at 7 T.

**Table 2:**
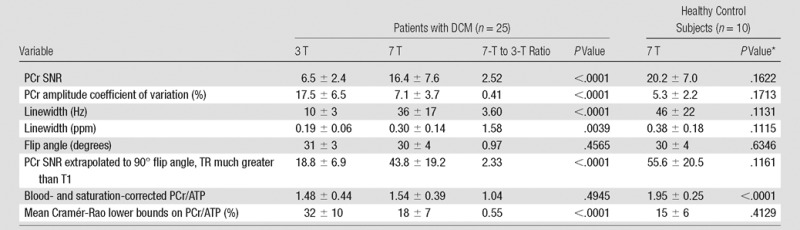
Comparison of Cardiac ^31^P Spectra Recorded in 25 Patients with DCMdilated cardiomyopathy at 3 T and 7 T and 10 Healthy Control Subjects at 7 T

Note.—Values are means ± standard deviation, unless otherwise indicated. ppm = parts per million, TR = repetition time, T1 = longitudinal (spin-lattice) relaxation time.

*Comparison of control subjects with patients with DCM dilated cardiomyopathy at 7 T.

**Figure 2:**
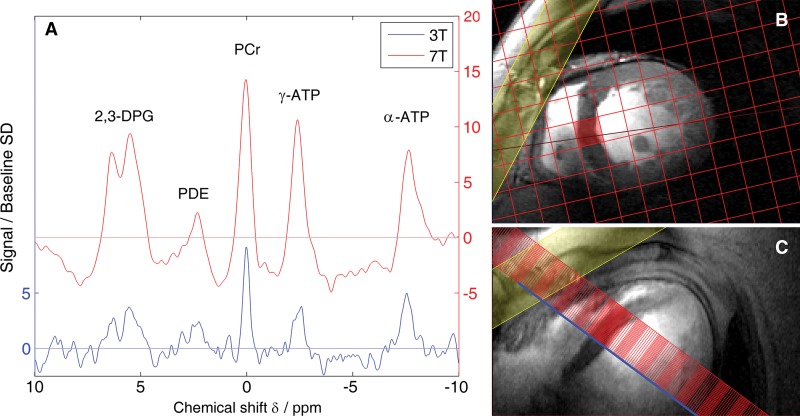
*A*, Graph shows comparison of spectra in a typical patient (57-year-old woman) at 3 T and 7 T. These spectra have had a matched filter applied and have been normalized to mean baseline noise, so the PCrphosphocreatine peak height is, by definition, the PCrphosphocreatine
SNRsignal-to-noise ratio. Increase in SNRsignal-to-noise ratio at 7 T is readily apparent. *B*, Corresponding mid-short axis localizer image acquired at 7 T. *C*, Corresponding four-chamber localizer image acquired at 7 T. The spectrocopy matrix is overlaid in red, and the voxel plotted in *A* is highlighted. The yellow-shaded region denotes the regional saturation slab used to suppress signal from overlying skeletal muscle.

Typical spectra for a patient with DCMdilated cardiomyopathy (Fig 2) show the increased SNRsignal-to-noise ratio at 7 T. The SNRsignal-to-noise ratio for PCrphosphocreatine was 2.5 times higher at 7-T field strength than at 3 T. Cramér-Rao lower bounds were 45% lower at 7 T than at 3 T, showing that the higher quality spectra obtained at 7 T enable more precise metabolite quantification (Fig 1b). Note, however, that the mean PCrphosphocreatine linewidth was higher at 7 T (36 Hz) than at 3 T (10 Hz). The 2.5 times higher SNRsignal-to-noise ratio in spite of this increase in linewidth (Fig 1c) suggests that using optimized per-subject B_0_ shim settings may further improve the quality of cardiac ^31^P MR spectroscopy at 7 T.

### Correlations of LV Functional Parameters with the PCr/ATP

The 7-T PCr/ATPphosphocreatine to adenosine triphosphate concentration ratio correlated with LVleft ventricle end-diastolic volume (*r* = −0.59, *P* = .0002), LVleft ventricle end-systolic volume (*r* = −0.60, *P* = .0001), LVleft ventricle ejection fraction (*r* = 0.51, *P* = .002), peak circumferential systolic strain (*r* = −0.44, *P* = .012), and peak diastolic strain rate (*r* = 0.38, *P* = .034). This suggests that, as remodeling parameters and mechanical function of the LVleft ventricle deteriorate, so does the myocardial energy deficit.

## Discussion

All participants imaged at 3 T also successfully completed the 7-T sequence, demonstrating that cardiac 7-T MR imaging and spectroscopy is well tolerated by patients. Cardiac ^31^P MR spectroscopy showed a 2.5 times increase in SNRsignal-to-noise ratio at 7 T compared with our best methods at 3 T. The PCr/ATPphosphocreatine to adenosine triphosphate concentration ratio was similar at both field strengths, excluding any new bias at 7 T. The Cramér-Rao lower bounds (measuring uncertainty) of PCr/ATPphosphocreatine to adenosine triphosphate concentration ratio showed a 2.2 times improvement at 7 T.

The higher SNRsignal-to-noise ratio at 7 T can be used *(a)* to obtain higher SNRsignal-to-noise ratio spectra, *(b)* to increase spatial resolution (eg, for investigating regional differences), or *(c)* to decrease the acquisition time (eg, to allow dynamic studies under more acute stress conditions than could be tolerated for a full 28-minute protocol). The increased precision (ie, decreased Cramér-Rao lower bounds) of PCr/ATPphosphocreatine to adenosine triphosphate concentration ratio also may aid separation of subject groups, either providing greater confidence in the difference between two groups or allowing the identification of smaller between-group differences.

As found in previous studies at lower field strengths, at 7 T the PCr/ATPphosphocreatine to adenosine triphosphate concentration ratio of patients with DCMdilated cardiomyopathy was significantly lower than that of control subjects (6,7,25). Although we did not acquire control data at 3 T for this study, previous work by our group has shown average PCr/ATPphosphocreatine to adenosine triphosphate concentration ratio in healthy control subjects to be 2.07 ± 0.38 (5), which would be significantly higher than the 3-T PCr/ATPphosphocreatine to adenosine triphosphate concentration ratio of 1.48 ± 0.44 in our patients with DCMdilated cardiomyopathy and which would be comparable to that of our 7-T control group (*P* = .37). Our 7-T results are also consistent with findings from lower-field ^31^P MR spectroscopic studies (6), showing that the PCr/ATPphosphocreatine to adenosine triphosphate concentration ratio correlates with the LVleft ventricle ejection fraction. We further observed that the correlations with the PCr/ATPphosphocreatine to adenosine triphosphate concentration ratio extended also to other markers of LVleft ventricle remodeling such as LVleft ventricle end-diastolic volume and LVleft ventricle end-systolic volume and more subtle earlier markers of LVleft ventricle dysfunction such as impaired peak systolic strain and impaired diastolic strain rates. These findings suggest that the increased precision of measuring PCr/ATPphosphocreatine to adenosine triphosphate concentration ratio at 7 T might improve the ability of phosphorus spectroscopy to deliver biochemical insights through comparison with other important cardiac parameters in future studies.

The mean ± standard deviation of PCr/ATPphosphocreatine to adenosine triphosphate concentration ratio in control subjects here was 1.95 ± 0.25 (7 T, control subjects, 16-element coil), which we can compare with 2.08 ± 0.33 (7 T, control subjects, 10 cm coil [8]) with 1.71 ± 0.48 (3 T, control subjects, 10 cmcoil [8]) and with 2.07 ± 0.38 (3 T, control subjects, heart-liver coil, average of three voxels [5]). The standard deviation of the PCr/ATPphosphocreatine to adenosine triphosphate concentration ratio decreases with increased field strength and with more sophisticated radiofrequency coils. This is consistent with increased measurement precision where the measurement precision is less than the biologic variability.

However, in patients with DCMdilated cardiomyopathy, we observed only a slight reduction in the standard deviation of the PCr/ATPphosphocreatine to adenosine triphosphate concentration ratio (1.48 ± 0.44 at 3 T vs 1.54 ± 0.39 at 7 T, *F* test *P* = .18). This suggests that the true biologic variability of PCr/ATPphosphocreatine to adenosine triphosphate concentration ratio in patients with DCMdilated cardiomyopathy might be greater than that in healthy volunteers and of a magnitude sufficient to contribute substantially to the observed scatter at 7 T. Authors of other studies have reported an increase in the standard deviation of the PCr/ATPphosphocreatine to adenosine triphosphate concentration ratio in patients with DCMdilated cardiomyopathy (1.41 ± 0.12) compared with control subjects (1.80 ± 0.06 [26]) and in those with severe DCMdilated cardiomyopathy (1.44 ± 0.52) compared with control subjects (1.95 ± 0.45 [7]) and of the mean PCrphosphocreatine concentration in patients with heart failure (8.3 ± 2.6) compared with control subjects (10.1 ± 1.3 [27]). Determining whether this is a real effect will require future studies, but if it is confirmed, then 7-T ^31^P MR spectroscopy could offer an enhanced ability to resolve disease-related variations in PCr/ATPphosphocreatine to adenosine triphosphate concentration ratio among patients and could allow better resolution to changes in response to therapy.

For this comparison, we chose to compare our most widely used 3-T coil (a heart-liver loop-butterfly coil with hardware quadrature combination, Siemens product for 1.5 T, retuned for 3 T) with our best available 7-T coil (a 16-element receive array used with a software combination and the whitened singular value decomposition algorithm [28]). The improvement seen here reflects real-world improvement at 7 T compared with 3 T. This is due both to the increase in field strength and to the optimized coil at 7 T. However, we note that when we previously attempted to introduce receive array coils at 3 T, we saw an increase in field of view for the coil, but with no statistically significant change in SNRsignal-to-noise ratio in the interventricular septum (actually a 6% loss in SNRsignal-to-noise ratio) compared with that with the use of the heart-liver coil (12). In practice, the differences in SNRsignal-to-noise ratio between coils optimized for cardiac applications at 3 T are typically 10%, which is much smaller than the gains due to increasing field strength from 3 T to 7 T (2.5 times increase in SNRsignal-to-noise ratio).

In this comparison, we chose to match flip angles at 30° in the interventricular septum at both 3 T and 7 T. This is slightly lower than the Ernst flip angle at both field strengths. By using T1 values from the literature (8) for ^31^P-containing metabolites, one can compute that this choice of flip angle accounts for approximately 8% of the PCrphosphocreatine
SNRsignal-to-noise ratio gain at 7 T.

### Limitations

Individual-subject 7-T to 3-T PCrphosphocreatine
SNRsignal-to-noise ratio ratios ranged from 0.68 to 6.56; this likely reflects differences in the coil-to-septum distance and in the loading of the two coils at 3 T and 7 T. Other groups have made similar observations (3,29).

The increased linewidth at 7 T relative to 3 T is likely due to the increased effect of different tissue magnetic susceptibilities at the higher field strengths (eg, at the heart-lung interface); optimized per-subject B_0_ shimming should be able to mitigate this effect in the future. Per-subject B_0_ shimming requires ^1^H imaging throughout the chest to measure B_0_ maps, followed by a shim current calculation and then cardiac ^31^P MR spectroscopy in the same sequence. This is possible and can give an approximately 20% decrease in the PCrphosphocreatine linewidth, but only with sophisticated hardware (30).

In comparison to our previous work using a 10-cm loop radiofrequency coil, the 28 × 30 cm^2^ transmit loop in the 16-element array coil (11) provided a more uniform excitation across the heart, but with a corresponding reduction in the peak B_1_^+^. This meant that we could not reliably excite β-ATPadenosine triphosphate in this study, whereas it was straightforward to do so using the 10-cm loop coil. We plan to upgrade our radiofrequency hardware in future to allow a uniform and high peak B_1_^+^.

We imaged patients in the prone position at 3 T as in previous studies, but we chose to image them in the supine position at 7 T. This was for two reasons: it improved patient comfort and it facilitated swapping between the ^1^H loop coil for localization and the ^31^P array for spectroscopy. In our experience, at 3 T, imaging prone versus supine makes no difference to the quality of ^31^P spectra in the interventricular septum. In a CT and MR imaging study of 16 patients (31), researchers observed no change in the position of the medial aspect of the heart, which would include our target voxel in the interventricular septum, although the anterior and lateral aspects of the myocardium moved anteriorly. In any event, if the heart did move in this way, it would have caused us to underestimate the potential gain in SNRsignal-to-noise ratio at 7 T.

At present, very few patient implants have been tested at 7 T, which excluded 40% of potential participants in this study. In order for 7-T MR imaging to be used in larger trials, or routinely in the clinic, widespread testing of common implants will be needed.

### Conclusions

Cardiac phosphorus spectroscopy is demonstrated to be feasible in patients at 7 T, giving higher SNRs and more precise quantification of the PCr/ATPphosphocreatine to adenosine triphosphate concentration ratio than at 3 T in a group of 25 patients with DCMdilated cardiomyopathy. These 7-T cardiac ^31^P MR spectroscopic methods provide a powerful tool that will enable us to better understand myocardial energetics, to identify differences in diseased tissue with greater confidence, or to perform studies in smaller populations than has been possible until now. For example, this technique will enable us to assess the effects of energy-sparing drugs in patients with DCM in a forthcoming clinical study.

**Advances in Knowledge**

■ Cardiac 7-T MR spectroscopy is feasible and well tolerated in patients.
■ The signal-to-noise ratio (SNR) of phosphorus spectroscopy in patients with dilated cardiomyopathy was 2.5 times higher at 7-T field strength (phosphocreatine SNR = 16.4 ± 7.6 at 7 T), compared with spectroscopy at 3-T field strength (phosphocreatine SNR = 6.5 ± 2.4 at 3 T).
■ The Cramér-Rao lower bounds in the uncertainty of metabolite quantification with phosphorus spectroscopy in the human heart were 45% lower at 7-T field strength compared with those at 3-T field strength (the percentage of phosphocreatine Cramér-Rao lower bounds decreased from 17.5% ± 6.5 at 3 T to 7.1% ± 3.7 at 7 T in patients with dilated cardiomyopathy).

**Implication for Patient Care**

■ The use of 7-T MR imagers for cardiac phosphorus spectroscopy allows more precise quantification of cardiac metabolites, which is an important step toward monitoring the metabolic state of an individual patient’s heart over time.
